# A Sluggish Atrioventricular  Node

**Published:** 2010-06-05

**Authors:** Tarun W Dasari, George Madden, Ralph Lazzara

**Affiliations:** 1 Section of Cardiovascular Medicine, University of Oklahoma Health Sciences Center, Oklahoma City, Oklahoma; 2 Department of Internal Medicine, University of Oklahoma Health Sciences Center, Oklahoma City, Oklahoma

**Keywords:** Prolonged PR interval, Wenckebach

##  Case Presentation

A 37 year old male with a history of peptic ulcer disease presented to the emergency room with nausea and vomiting. He reported two recent episodes of syncope, but denied other associated symptoms including palpitations, hematemesis, melena, chest pain, or dyspnea. He reported recent use of marijuana, but denied use of cocaine or methamphetamines. The patient reported similar symptoms in 2006 when he was diagnosed with Helicobacter pylori associated peptic ulcer disease.

**Figure 1:**
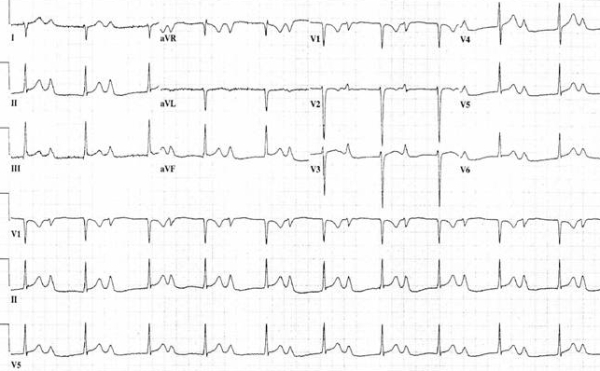
Showing extreme prolongation of PR interval, biatrial enlargement (tall prominent P wave in II and wide negative deflection of the p wave in V1).

**Figure 2:**
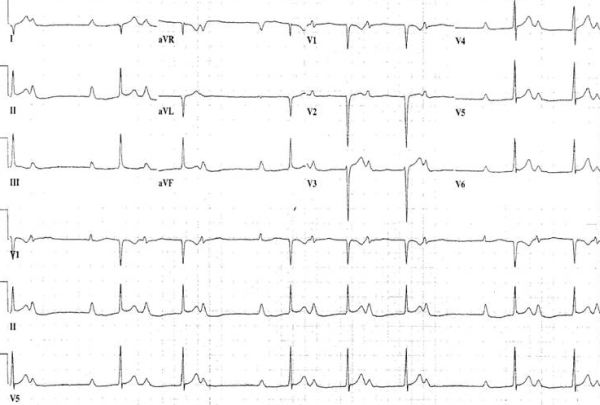
Showing Mobitz II block in addition to 1st degree AV block.

Upon presentation, he was in no apparent distress and his physical exam was unremarkable. He was afebrile with heart rate of 68 beats/min, blood pressure 128/72 mm Hg, and respiratory rate was 14/minute with 100% oxygen saturation on room air. Incidentally, his 12 lead electrocardiogram (ECG) on presentation showed normal sinus rhythm but an extreme prolongation of PR interval of up to 690 m-sec, signs of biatrial enlargement and right axis deviation (Figure 1). A repeat ECG the next day revealed Wenckebach (Mobitz Type I) phenomenon (Figure 2). Upon review of his old ECGs, he did have evidence of 1st degree AV block but to a lesser degree (PR interval of 300 msec). His cardiac biomarkers showed a troponin I of 4.2 ng/ml (normal < 0.39 ng/mL) and Creatinine Kinase-MB of 7.1 ng/ml (normal <4.9 ng/mL).  He was admitted for further work up to evaluate the severe prolongation of PR interval and elevated cardiac enzymes.

Serial cardiac biomarkers trended down to normal within 24 hours. Urine drug screen was positive for marijuana. Upon review of his medical records over the last 3 years, he had a normal coronary angiogram and asymptomatic treadmill stress testing achieving 90% of his target heart rate and exercising for 14.5 minutes on the Bruce protocol, with no further worsening of his PR interval with exercise. Our goal in ordering subsequent diagnostic studies was to determine the etiology of his conduction delay and to rule out arrhythmogenic causes of syncope.

**Figure 3:**
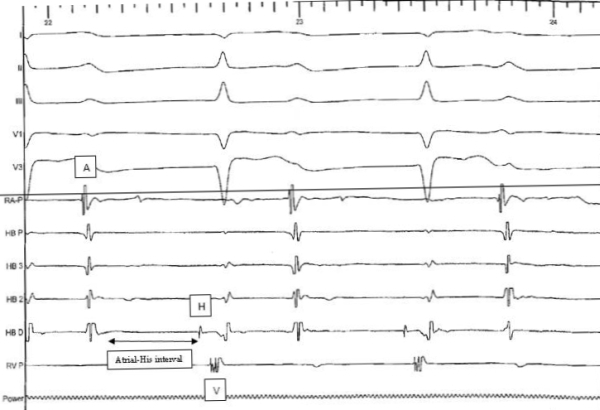
Invasive electrophysiology tracing of atrium (A), his bundle (H) and ventricle (V) demonstrating a significant delay in atrial-his conduction (490 msec).

Transthoracic echocardiogram which revealed mild right atrial enlargement, normal left and right ventricular function and mild focal outpouching of right ventricular apex. There was no evidence of diastolic dysfunction. He subsequently underwent a cardiac MRI which showed no evidence of fibro fatty myocardial involvement and mild hypokinesis of the right ventricular free wall. To better characterize the first degree AV (atrioventricular) block and to rule out right ventricular inducible tachycardia we elected to proceed with an electrophysiological study. Baseline study showed an atrial-His interval of 490 msec (normal upto 130 msec) and a normal His-ventricular interval of 35 msec (Figure 3). This proved that the predominant conduction delay was within the AV node with no evidence of intra atrial conduction delay. We were also able to induce monomorphic ventricular tachycardia easily with triple extra stimulation. An endomyocardial biopsy, done to rule out infiltrative disease processes as a cause of his conduction problems, was negative for amyloidosis and arrhythmogenic right ventricular dysplasia, although the sensitivity and negative predictive value of endomyocardial biopsy is low in ruling out such disease processes.

## Management and Discussion

The exact etiology of a diseased AV node was not determined in this patient. Atrial systole was occurring in close proximity to the preceding ventricular systole, which could have resulted in atrial contraction prior to complete atrial filling and possibly against a closed atrioventricular valve. The result could be a compromise in ventricular filling, an increase in pulmonary capillary wedge pressure, and a decrease in cardiac output leading to symptoms of dizziness and syncope. Our patient could have had higher degrees of AV block which by itself could lead to syncope. Hence due to extreme 1st degree AV block and intermittent Wenckebach coupled with the inducible ventricular tachycardia, in a patient with 2 episodes of syncope, we elected to implant a dual chamber pacemaker/defibrillator. Such degree of PR interval prolongation is unusual and may be under reported in the literature. A mild degree of PR prolongation can occur as a normal variant in healthy young individuals without apparent heart disease [1,2]. It can also occur with the use of agents such as beta-blockers, calcium channel blockers and digoxin, and increased vagal tone such as in young healthy atheletes. The long PR interval can either be due to increased atrial -His conduction (represented by A-H interval) or increased His-ventricular conduction time. Increased PR interval is usually secondary to AV nodal disease (narrow QRS) and infranodal disease contributes to a minority (broader QRS complex). The aforementioned PR prolonging drugs may be avoided in such cases. There is no definite threshold for the PR interval to avoid such drugs as long as the patient is asymptomatic. We tend to avoid such drugs when patients have symptoms of poor perfusion even with first degree AV block. The implantation of pacemaker is controversial in this setting and the ACC/AHA/ESC 2008 guidelines give a Class IIa (level of evidence B) recommendation for permanent pacemaker in symptomatic 1st degree AVB [3]. Endomyocardial biopsy is not a sensitive test to rule out causes of infiltrative cardiomyoapthies. Our patient had right atrial enlargement and conduction abnormalities. There might be possibility of development of restrictive cardiomyopathy and will require a close follow up. Familial AV block is also a reported cause of AV block and is associated with progression of first degree AV block to second degree blocks in young adulthood and may be associated with cardiomyopathy [4].

In conclusion, extreme degrees of PR interval prolongation can occur and often indicate a diseased AV node. In such cases it is prudent to avoid beta blocker and calcium channel blockers, especially when symptoms of poor perfusion such as dizziness and syncope are present. Routine use of permanent pacemakers for isolated first degree AV block in asymptomatic patient is not indicated but can be considered in those with symptoms.

## References

[R1] Johnson RL (1960). Electrocardiographic findings in 67,375 asymptomatic subjects. VII. Atrioventricular block. Am J Cardiol.

[R2] Viitasalo MT (1982). Ambulatory electrocardiographic recording in endurance athletes. Br Heart J.

[R3] Epstein AE (2008). ACC/AHA/HRS 2008 Guidelines for Device-Based Therapy of Cardiac Rhythm Abnormalities: a report of the American College of Cardiology/American Heart Association Task Force on Practice Guidelines. Circulation.

[R4] Fernandez P (2004). Progressive familial heart block type II (PFHBII): a clinical profile from 1977 to 2003. Cardiovasc J S Afr.

